# Exposure to musculoskeletal and psychosocial risks in SME workers, Antofagasta, Chile, 2021-2022

**DOI:** 10.47626/1679-4435-2023-1119

**Published:** 2024-11-14

**Authors:** Guido Solari Montenegro, Monserrat Rivera Iratchet, Alejandra Velasco Mur, Milena Álvarez Andrade, Karin Orellana Urra, Paola Gómez Inostroza

**Affiliations:** 1 Department of Rehabilitation Sciences, Universidad de Antofagasta, Facultad de Ciencias de la Salud, Antofagasta, Chile; 2 Department of Occupational Therapy and Speech Therapy, Universidad de Antofagasta, Facultad de Ciencias de la Salud, Antofagasta, Chile

**Keywords:** occupational health, job satisfaction, public health, salud ocupacional, satisfacción en el trabajo, salud pública

## Abstract

**Introduction:**

In Chile, small and medium-sized enterprises employ approximately 5,000,000 workers, of whom only 10% are formally protected from accidents and occupational diseases, and there is little information on their exposure to occupational hazards and demographic characteristics.

**Objectives:**

To identify some occupational musculoskeletal and psychosocial risks and sociodemographic characteristics in small and medium-sized enterprises workers in the region of Antofagasta, Chile.

**Methods:**

Using a descriptive and exploratory design, their sociodemographic characteristics and the jobs of a sample of 273 workers were evaluated to determine the risks due to manual handling of loads, work-related upper extremity musculoskeletal disorders, and psychosocial factors. A sociodemographic survey, the technical guide of the Ministry of Labor, the technical standard and checklist of the Ministry of Health, and the Superintendencia de Seguridad Social/Instituto Sindical de Trabajo, Ambiente y Salud questionnaire of the Superintendency of Social Security were used.

**Results:**

Overall, 23% of workers presented normal body mass index, 11.7% were at risk for manual handling of loads, and 16.9% at risk for work-related upper extremity musculoskeletal disorders. The greatest prevalence of psychosocial risk was observed in the double presence dimension (46.89%). A relationship was only found between psychological demands and compensation dimensions and the job positions and between psychological demands and economic activity.

**Conclusions:**

Risk for manual handling of loads and work-related upper extremity musculoskeletal disorders was found in mining service companies, in men with technical and blue-collar positions, and in those with high psychosocial risk in the double presence dimension. An association was identified between two psychosocial dimensions with the workers’ positions and the economic activity of their company.

## Abstract (es)

**Introducción:**

En Chile las pequeñas y medianas empresas (Pymes) emplean aproximadamente 5.000.000 trabajadores, el 10% de los cuales está protegido de accidentes y enfermedades laborales, y existe escasa información sobre su exposición a riesgos laborales y características demográficas.

**Objetivos:**

Identificar algunos riesgos laborales musculoesqueléticos, psicosociales y características sociodemográficas en trabajadores de pequeñas y medianas empresas de la región de Antofagasta, Chile.

**Métodos:**

Mediante un diseño descriptivo y exploratorio, se identificaron las características sociodemográficas de 273 trabajadores y se evaluaron sus puestos de trabajo para determinar los riesgos por manejo manual de cargas, por trastornos musculo esqueléticos de extremidades superiores relacionados al trabajo y por factores psicosociales. Se utilizó una encuesta sociodemográfica, la guía técnica del Ministerio del Trabajo, la norma técnica y la lista de chequeo del Ministerio de Salud, y el cuestionario de la Superintendencia de Seguridad Social/Instituto Sindical de Trabajo, Ambiente y Salud de la Superintendencia de Seguridad Social.

**Resultados:**

El 23% de los trabajadores presentó índice de masa corporal normal, 11,7% tenía riesgo de manejo manual de cargas y el 16,9% tenía riesgo de trastornos musculoesqueléticos de extremidades superiores. La mayor prevalencia de riesgo psicosocial alto se agrupó en la dimensión doble presencia (46,89%). Se encontró relación entre las dimensiones exigencias psicológicas y compensaciones con los cargos y entre exigencias psicológicas y actividad económica.

**Conclusiones:**

Se halló riesgo de manejo manual de cargas y trastornos musculo esqueléticos de extremidades superiores en empresas de servicios a la minería, en hombres con cargos técnicos y operarios, y en aquellos con riesgo psicosocial alto en la dimensión doble presencia. Se identificó asociación entre dos dimensiones psicosociales con los cargos de los trabajadores y la actividad económica de su empresa.

## INTRODUCTION

The term SME refers to small and medium-sized enterprises that have from 10 to 49 employees (small-sized enterprises) and from 50 to 199 employees (medium-sized enterprises)^1^ and/or whose annual sales are from 2,400 Chilean Development Units (Unidades de Fomento, UF) to 100,000 UF per year.^2^ Most SMEs of countries belonging to the Organisation for Economic Co-operation and Development (OECD) are small or medium-sized, account for approximately 60% of total employment, and play an important role in economic growth, job creation, local/regional development, and social cohesion.^2,3^

In Chile there are approximately 235,569 SMEs, which provide formal employment to more 5,000,000 workers in the country, but only 10% of these enterprises are affiliated to an Administrative Body of Law no. 16,744 (*Organismo Administrador de la Ley* 16,744, OAL). Therefore, 90% of their workers are not protected against possible situations of necessity derived from occupational accidents and diseases and do not gain any benefit from actions implemented by OALs to protect their health and safety.^4^

However, risk management and knowledge of insurance against occupational accidents and diseases are very important to workers’ quality of life, and there is evidence that employers and employees have little knowledge about Law no. 16,744 about the importance of occupational risk management in their companies.^5^

In terms of health, musculoskeletal disorders consist of a relevant national problem, particularly in male and young workers and in the areas of industry, transportation, agriculture, fishing, commerce and construction, and women have the highest complaints in the areas of personal services, commerce, and financial and professional services. Problems related with psychosocial factors have also been reported as relevant problems among Chilean workers,^6^ since they have a strong influence of workers’ health. Such psychosocial factors derive from interactions between work, environment, job satisfaction, organizational conditions, and workers’ capacities (their needs, their culture, and their personal situation).^7^ Evidence exists that such factors can positively affect workers’ well-being, or negatively affect people’s health.^8^

Nevertheless, it is necessary to generate additional information to rationally plan management, promotion, and prevention of health risks and safety of SME workers in the region of Antofagasta.

### OBJECTIVES (RESEARCH QUESTION)

What are the sociodemographic characteristics and musculoskeletal risks associated with manual handling of load (MHL), risks for repeated upper extremity musculoskeletal disorders, and psychosocial risks in a sample of SME workers in the region of Antofagasta, northern Chile?

## METHODS

A descriptive exploratory study was conducted from March 2021 to August 2022 (period of the COVID-19 pandemic) to assess a random, non-probabilistic, convenience sample of 273 workers (153 men and 120 women), including all who were available (with their consent and considering their workload) considering the overall number of SMEs that agreed to participate and that operated in the region of Antofagasta, Chile.

The long duration of data collection was related to the periods of physical isolation that should be followed by the researchers (and some workers), in compliance with the recommendations from the Ministry of Health to control the COVID-19 pandemic. Workers’ labor conditions and the tasks they performed were essentially the same as those of non-pandemic period. Participants should meet the following inclusion criteria: being a SME worker (either man or woman), being aged from 18- to 65-year-old, having a current employment contract, and having signed the informed consent form. Exclusion criterion was refusing to answer the surveys or being evaluated and/or observed (photographed, filmed) while working.

The researchers investigated four communes of the region of Antofagasta (Mejillones, Tocopilla, Calama, Taltal, and Antofagasta) and established contact with several SMEs according to economic activity, commune, and availability. Subsequently, the researchers informed study goals and methodology to the chief manager of each SME. Once approval for inclusion of each company in the study was obtained, their history, organizational structure, descriptions of jobs and positions, and previous reports on ergonomic evaluations, industrial hygiene, and occupational health assessed, and researchers’ visits were scheduled and coordinated.

Workers participated voluntarily by signing a consent form that was reviewed and approved by the Scientific Research Ethics Committee of Universidad de Antofagasta under folio number: 175/2019. Each worker was asked about written information on some of their sociodemographic characteristics and had their height and body weight measured using a stadiometer and a Seca scale, model 703, with internet connection. Finally, their body mass index (BMI) was calculated and interpreted according to the Garrow scale.^8^

A brief sociodemographic survey was administered to collect some personal information (age, sex, educational level, weight, height), and the risk for MHL was determined using the technical guide by the Chilean Ministry of Labor and Social Security.^9^ Furthermore, risk factors for work-related upper limb musculoskeletal disorders (WRMSDs-ULs) were identified and evaluated using the technical standard for identification and evaluation of risk factors related to WRMSDs-ULs created by the Chilean Ministry of Health^10^; finally, psychosocial risks were identified and evaluated using the short version of the Superintendencia de Seguridad Social/Instituto Sindical de Trabajo, Ambiente y Salud (SUCESO ISTAS-21) questionnaire.^11^

The SUSESO ISTAS-21 questionnaire employs a Likert-scale to investigate five dimensions (psychological demands, active labor and skills development, social support and leadership quality, compensation, and double presence) and classifies the level of psychological risk into three categories (high, medium, low). For its administration, the authors instructed workers on the purpose of the questionnaire and the appropriate procedure to complete it. Considering the total of workers, the prevalence of those with low, medium, and high risk was identified in each dimension. Questionnaire reliability as measured by the Cronbach’s alpha coefficient was estimated at 0.83.

Field assessments of MHL and WRMSDs-ULs were performed by a trained investigator, who analyzed job positions through direct observation and technical support of videos and photographs and assessed musculoskeletal risks for MHL and WRMSDs-ULs according to the categories proposed by instruments (no risk - white, presence of risk - yellow, critical condition - red).

In the case of MHL, the technical guide was applied to all job positions that involve manual handling of loads above 3 kg. The application of this guide included the initial application of a simple methodology (such as the Manual Handling Assessment Charts [MAC]^12^) and occasionally of an advanced methodology (such as the National Institute for Occupational Safety and Health [NIOSH] *lifting equation* or *lifting index*)^13^ when the working procedures were more complex. Risk assessment of WRMSDs-ULs was performed exactly as indicated in the procedure established in the technical standard. All information obtained was stored on a spreadsheet, based on which descriptive information of the variables was generated.

## RESULTS

In terms of sociodemographic variables, the majority of the workers evaluated were men (56%), and most of the sample was aged from 26 to 45 years of age. In both sexes, the predominant education level was secondary education, either scientific-humanistic or technical vocational (66%), followed by elementary education (22%), and higher education (12%). As for the working days of the week, most participants worked from Monday to Saturday (95%), and their working hours convers mainly the morning and afternoon shifts.

Regarding population’s anthropometric characteristics, in terms of BMI, only 23% of the sample was classified as normal weight, and the remaining workers in the sample were classified, in a descending order, as overweight (42%), obese (33%), and morbidly obese (2%). It was observed that the percentage of women with normal weight (20%) was lower than that of men (25.5%).

Regarding risk for MHL, 32 workers (11.7%) were at risk for MHL. When these workers were divided according to their economic activity, 2 worked in the transportation sector, 24 in mining service sector, and 6 in the commerce sector. With regard to position, half of workers at risk were blue-collar workers (n = 16), and the other half consisted of technicians (n = 16). No job position was shown to be associated with critical risk condition (or red risk) for MHL (Table 1).

**Table 1:** Identification of economic activity and job positions at risk for MHL in the study sample (n = 273), Chile, 2021

Economic activity of the enterprise	Job category	Job title	At-risk workers by position		Risk condition for MHL^*^		
			n	%	No risk	Presence of risk or yellow risk	Critical risk condition or red risk
Transportation	Blue-collar worker (2)	Tire mechanic	2	0.7		x	
Mining services	Administrative/technician (1)	Quality control and metal-mechanic process manager	1	0.4		x	
	Technician (15)	Metal-mechanic quality control operator	3	5.5		x	
		Metal-mechanic electrician on duty	2			x	
		Metal-mechanic operator	3			x	
		Specialist welder	7			x	
	Blue-collar worker (8)	Metal-mechanic operator assistant	5	2.9		x	
		Warehouse manager	3			x	
Comercio	Blue-collar worker (6)	Yard foreman at an industrial hardware store	2	2.2		x	
		Warehouse manager at a supermarket	1			x	
		Multifunctional assistant at an industrial hardware store	3			x	
Total workers presenting risk for MHL			32	11.7			
Total workers evaluated in the study			273				

MHL = manual handling of loads.

* According to the Chilean Ministry of Labor and Social Security.^8^

With regard to risk for WRMSDs-ULs: Overall, 46 workers were found at risk for WRMSDs-ULs (16.9% of all workers assessed). When the number of workers was divided into the economic activity of the company where they work, 15 of them were engaged in the transportation sector, 37 in the mining services sector, and 4 in the commerce sector. In relation to position or occupation, most study participants were technicians (27), followed by blue-collar workers (19), with both groups together accounting for 16.9% of total workers evaluated in this study There was no job position associated with critical risk condition (or red risk) for WRMSDs-ULs (Table 2).

**Table 2:** Identification of economic activities and job positions at risk for WRMSDs-ULs in the study sample (n = 273), Chile, 2021

Economic activity of the company	Job category	Job title	At-risk workers by job position		Risk condition for WRMSDs-ULs^*^		
			n	%	No risk	Presence of risk (yellow)	Critical risk condition (red)
Transportation	Blue-collar worker (15)	Bus janitor	11	5.5		x	
		Tire mechanic	4			x	
Mining services	Technician (27)	Production operator at a metal-mechanic plant	14	9.9		x	
		Metal-mechanic welder	7			x	
		Metal-mechanic operator	3			x	
		Warehouse clerk	3			x	
Commerce	Blue-collar worker (4)	Manufacturer of cocktail products	2	1.5		x	
		Dishwasher at a canteen	2			x	
Total workers at risk for WRMSDs-ULs			46	16.9			
Total workers evaluated in the study			273				

WRMSDs-ULs = work-related upper limb musculoskeletal disorders.

* According to the Chilean Ministry of Health.^9^

With regard to psychosocial factors, considering the overall sample (273), the prevalence of workers with low, medium, and high risk for each dimension was obtained. The greatest prevalence of high level of risk was observed in the double presence dimension (46.89%), followed by social support and leadership quality (36.27%) and compensation (32.24%) dimensions. In relation to low level of risk, the highest prevalence was found in the psychological demands and active work and skills development dimensions (Table 3).

**Table 3:** Distribution of level of psychosocial risk by dimensions and sex in the study sample (n = 273), Chile, 2021

Risk dimensions	Level of risk according to sex^*^											
	Low				Medium				High			
	Men		Women		Men		Women		Men		Women	
	n	%	n	%	n	%	n	%	n	%	n	%
Psychological demands	56	20.52	61	22.34	51	18.68	32	11.72	46	16.85	27	9.89
Active labor and skills development	52	19.04	45	16.49	56	20.51	44	16.12	45	16.49	31	11.35
Social support and leadership quality	39	14.29	42	15.38	59	21.62	34	12.46	55	20.14	44	16.11
Compensation	42	15.39	47	17.22	56	20.52	40	14.65	55	20.14	33	12.08
Doble presence	31	11.35	27	9.89	52	19.05	35	12.80	70	25.64	58	21.24

* 153 men and 120 women.

† According to the Superintendencia de Seguridad Social/Instituto Sindical de Trabajo, Ambiente y Salud (SUSESO ISTAS-21) questionnaire.^10^

In relation to sex, 25.64% of men were classified as having high risk for double presence, followed by an equal percentage (20.14%) of high risk for social support and compensation dimensions. Among women, the prevalence of high risk was greater (21.24%) in the double presence dimension, followed by social support and compensation (16.11 and 12.08%, respectively).

The double presence dimension presented the greatest prevalence of high risk in all job positions (Table 4).

**Table 4:** Percent distribution of levels of psychosocial risk according to positions and economic activities in a sample of SMEs (n = 273), Antofagasta, Chile, 2021

Risk dimensions	Job category									Economic activity of the company								
	Level of risk^*^									Level of risk^*^								
	Blue-collar workers			Technicians			Administrative workers			Transportation			Mining services			Commerce		
	H	M	L	H	M	L	H	M	L	H	M	L	H	M	L	H	M	L
Psychological demands	20.0	27.0	53.0	26.7	40.0	33.3	48.3	31.0	20.7	30.7	23.1	46.2	34.1	29.5	36.4	15.2	31.3	53.5
Active labor and skills development	26.5	37.7	35.8	30.0	40.0	30.0	27.0	31.0	42.0	26.9	42.3	30.8	34.1	35.2	30.7	20.2	38.4	41.4
Social support and leadership quality	37.8	31.7	30.5	33.3	16.7	20.0	41.4	31.0	27.6	57.7	23.1	19.2	33.0	39.8	23.9	32.3	32.3	35.4
Compensation	31.1	33.8	35.1	36.7	36.7	26.6	31.0	41.4	27.6	53.8	38.5	7.7	37.5	38.6	23.9	21.2	32.3	46.5
Double presence	49.6	30.5	19.9	53.3	30.0	16.7	55.2	34.5	10.3	46.2	34.6	19.2	52.3	33.0	14.7	50.5	29.3	20.2

SMEs = small and medium enterprises.

* Level of risk: H = High level of risk; M = Medium level of risk; L = Low level of risk.

† According to the Superintendencia de Seguridad Social/Instituto Sindical de Trabajo, Ambiente y Salud (SUSESO ISTAS-21) questionnaire.

The dimension with the second greatest prevalence of high risk was psychological demands among administrative workers, with a prevalence of 48.3%; compensation among technicians, with a prevalence of 36.7%; and social support and leadership quality among blue-collar workers, with a prevalence of 37.8%.

With regard to the risk for MHL according with the economic activity of the company and psychosocial risk dimensions, it was observed that the double presence dimension presented the higher prevalence of people (average of 50%) with high risk for MHL in the three categories of economic activity. The highest prevalence of low risk for MHL (average of 40%) - in the three categories of economic activity - was observed in the following dimensions: psychological demands and active work and skill development.

## DISCUSSION

Similar to the reported by other authors,^14^ most of the population in our study consisted of men (56%) and had scientific-humanist or professional vocational education (66%), which the importance given by the labor market to qualified workers, a finding coherent with reports of other Latin American countries and with a similar development than Chile.^15^

In relation to BMI values, the present article showed that approximately 65% of the population were overweight and obese, a percentage similar to that observed by other authors^16^ and that supports the convenience of promoting healthy lifestyles and greater efforts directed to foster health control so as to prevent non-communicable chronic diseases, which represent a major morbidity problem in the Chilean public health system.^17^

Our results show that - of the 273 workers - 32 were at risk for MHL and, according to ergonomic assessments of workplaces, this risk is mainly related to the combination between working position or posture (trunk flexion and torsion) and the necessary strength to perform their tasks, aspects that coincide with those reported by other authors. Furthermore, in terms of BMI and risk for MHL, it is necessary to consider that some authors have described an association between high BMI and increased risk for MHL and low back pain at work.^18,19^

Most workers at risk for WRMSDs-ULs were technicians or workers who predominantly used manual tools with repetitive movement patterns, a situation in line with that published in the systematic review by da Costa & Vieira,^20^ in which the greater frequency of upper limb musculoskeletal problems is associated with repetitive motion. Regarding this topic, other authors report that the back and the lower limbs are also loaded during repetitive tasks of the upper limbs, generating diffuse symptoms or in several body parts.^21,22^

In light of the foregoing, and considering the risks according to workers’ working position or posture, strength, and repetitive motion, the authors of the present study developed three documents for the SMEs: one of them with specific ergonomic recommendations for works at risk for WRMSDs-ULs and/or MHL; another document with recommendations for ergonomics self-adjustment in administrative tasks; and finally a guideline with compensatory exercises for people whose work involves upper limb repetitive movements. The documents were made available and shared with the risk prevention manager at each company.

In relation to psychosocial factors, similarly to Güilgüiruca et al.,^16^ the present study found a greater prevalence of high risk in the double presence dimension. Conversely, Acevedo et al.^23^ assessed a sample of 292 workers at an energy company and reported a greater percentage of female workers with high risk for the double presence dimension (21.2%); conversely, in our study there was a higher of men (25.6%) at high risk for the same dimension. However, it should be considered that a high level of risk for double presence is an expression of hardly compatible demands between work and personal/family spheres.

In the present study, the greatest prevalences of high risk were observed in double presence, social support and leadership quality; therefore, it is relevant to consider these aspects in order to prevent consequences both for organizations and for workers’ health.

Considering the three categories of economic activity, 50% of workers at risk for MHL simultaneously presented higher prevalence of high risk for the double presence dimension.

Considering the three categories of economic activity studied, 40% of workers with low risk for MHL simultaneously presented higher prevalence of low risk for the psychological demands dimension (α below 0.05) and active labor and skill development dimension. In a logical manner, the first finding implies that the effort workers make is low, or that the workers’ emotional or psychological demands are low, whereas the latter finding implies that workers have high control over their tasks, or that they are relevant for these workers and/or present with high possibilities of learning. The present study was conducted over a long time (17 months), because its approval and provision of means and resources to start its operational phase coincided with the COVID 19 pandemic, which significantly limited investigators’ field work, due to the need for geographic displacement and to the difficulty in having time access to SMEs.

## Conclusion

The results obtained showed that workers of the studied SMEs had an educational level compatible with qualified workers and that only 23% of them had normal weight, with the remaining workers potentially being at risk for metabolic changes that require special attention. Moreover, nearly 12 to 17% of workers are exposed to risk for MHL and WRMSDs-ULs, particularly in technical and operational positions and in mining service companies. Most workers presented with psychosocial factors particularly related to the double presence dimension in all job positions, in mining services, with a greater prevalence in men. The foregoing highlights the importance of approaching educated workers so as to promote healthy behaviors in terms of diet and physical activity and in terms of preventing the risk for MHL and WRMSDs-ULs.

However, the priority of occupation health should be given only to workers’ good habits and lifestyles, since it does not represent a neither responsible nor coherent management in occupational health. An important step to continue is promoting the dissemination and the participative collective analysis of study results, in order to put them in the context of each company in order to manage measures to change or mitigate risks by incorporating all necessary measures.^24^ The authors share the convenience of broadening the present study and contributing to public health from the occupational health point of view, with the generation of an extensive diagnosis that makes it possible to rationally manage the necessary means to improve workers’ wellbeing and the productivity of the companies where they work.
